# Diagnostic Imaging Studies on Local and Systemic Erythropoietin Application for Promoting Bone Regeneration in Rat Calvarial Defects

**DOI:** 10.3390/vetsci9100578

**Published:** 2022-10-18

**Authors:** Tsvetan Chaprazov, Radina Vasileva, Kiril Atliev, Elena Firkova

**Affiliations:** 1 Department of Veterinary Surgery, Faculty of Veterinary Medicine, Trakia University, 6000 Stara Zagora, Bulgaria; 2 Department of Urology and General Medicine Medical, Faculty of Medicine, Medical University of Plovdiv, 4000 Plovdiv, Bulgaria; 3 Department of Periodontology and Oral Diseases, Faculty of Dental Medicine, Medical University of Plovdiv, 4000 Plovdiv, Bulgaria

**Keywords:** erythropoietin, bone defect, bone regeneration, calvaria, rat

## Abstract

**Simple Summary:**

Erythropoietin (ЕPО) is a glycoprotein hormone which, apart from its physiological function in the regulation of erythropoiesis, has additional functions in the animal and human body. In orthopaedic patients EPO treatment induces regeneration of bone defects, proliferation of osteoblasts and new blood vessel formation, improvement of early endochondral ossification and bone mechanical strength, thus resulting in enhanced bone healing. The purpose of this study was to compare the effects of local and systemic application of recombinant human erythropoietin (rhEPO) on the healing of rat calvarial defects. The systemic effect of EPO treatment was monitored by haematological analyses on days 0, 30 and 90. Bone healing was monitored via radiography and computed tomography on the same time intervals. The results demonstrated that intraperitoneally-injected rhEpo did not result in bone formation. Simultaneously, it affects the erythropoiesis by increasing the erythrocyte counts, haemoglobin and haematocrit. At the same time, single local dose rhEpo applied on collagen carrier could be used for enhancing bone healing without systemic effect.

**Abstract:**

The purpose of this study was to compare the effects of local and systemic application of recombinant human erythropoietin (rhEPO) on the healing of rat calvarial defects. Twenty-four male skeletally-mature Wistar rats were used. Two bone 5 mm critical size defects were created in calvarial bones of each rat. In rats from experimental group I (*n* = 12), EPO was applied locally on a collagen cone in left defects, whereas a collagen cone soaked with physiological saline was placed in right defects. The rats from experimental group II were injected once intraperitoneally with 4900 IU/kg EPO; a collagen cone was only placed in left defects, whereas the right defects were left empty. The systemic effect of EPO treatment was monitored by haematological analyses on days 0, 30 and 90. Bone healing was monitored via radiography and computed tomography on the same time intervals. The results demonstrated that local EPO application had no significant effect on haemopoiesis, unlike the systemic application. At the same time, it resulted in new bone formation and therefore, could be successfully used as a means of promoting bone regeneration.

## 1. Introduction

The major part of bone defects heal spontaneously under an appropriate physiological environmental conditions due to the regeneration ability of bone tissue [1,2]. Bone healing requires cell components, signaling molecules, cytokines, growth factors and adequate blood supply [3]. The absence or impairment of some of these factors could result in delayed or non-union [4,5]. In these cases, as well as in the presence of large bone defects, bone regeneration should be assisted to achieve bone repair [6].

Erythropoietin (EPO) is a glycoprotein hormone which, apart from its physiological function in the regulation of erythropoiesis, has additional functions in the animal and human body [7,8,9,10,11]. Data about its osteogenic and angiogenic potential have raised the interest of researchers developing new strategies for the promotion of bone regeneration [12,13,14,15].

Previous experimental studies have demonstrated that erythropoietin treatment induces regeneration of bone defects [16,17,18], proliferation of osteoblasts [19,20], new blood vessel formation [12,21,22], improvement of early endochondral ossification and bone mechanical strength, thus resulting in enhanced bone healing [13]. In addition, clinical research in patients with tibiofibular fractures reported a two-week shorter mean duration in fracture repair, and better quality of bone healing, when treated with EPO injection [23]. Topically-administered EPO appears to be a novel preventive and therapeutic agent in bisphosphonate-related osteonecrosis of the jaw in humans [24]. The positive effect of EPO is assumed to be due to its angiogenic [25], anti-inflammatory [26] and anti-apoptotic [27] properties. The calvaria critical-size defect is an extensively-used model in bone tissue engineering due to its ability to create standardized defects for testing the osteogenic capacity of different biomaterials [28]. There is still controversy regarding whether local or systemic administration of EPO has a better bone repair capacity. The purpose of this study, therefore, is to compare the effects of systemic and single local dose recombinant human erythropoietin (rhEPO) on bone healing, using a rat calvarial critical-size defect model.

## 2. Materials and Methods

### 2.1. Animals

Twenty-four male skeletally-mature Wistar albino rats, 6 months of age, with average weight 262 ± 33 g were used in the experiments. The rearing and housing of experimental animals was fully compliant with conditions stipulated by Ordinance 20 of 1 November 2012 on the minimum requirements for protection and welfare of experimental animals and site requirements for use, rearing and/or their delivery, transposed from Directive 2010/63/EC. The experiment was performed in accordance with permit No. 241 issued by the Ethics Committee to the Bulgarian Food and Safety Agency.

### 2.2. Materials

Erythropoietin: Binocrit (Sandoz GmbH, Kundl, Austria) was used in this study. Binocrit 2000 IU is an injection solution, each mL containing 2000 IU epoietin alpha equivalent to 16.8 μg/mL. It was produced by recombinant DNA technology in Chinese Hamster Ovary cell line expression system.

Collagen sponge: Collacone (Botiss biomaterials GmbH, Berlin, Germany) is a collagen cone on the basis of porcine collagen. Its complete resorption occurs within 2–4 weeks.

### 2.3. Surgical Procedure

The rats were anesthetized with 80 mg/kg ketamine hydrochloride 10% (Anaket^®^, Richter Pharma AG, Wels, Austria) and 10 mg/kg xylazine hydrochloride 2% (Xylazin^®^, Bioveta, Ivanovice na Hané, Czech Republic) applied intramuscularly.

The surgical field was aseptically prepared. Hair was clipped, and the skin disinfected with 10% povidone-iodine (Braunol, B. Braun Melsungen AG, Melsungen, Germany).

A skin incision was created from the nasal bone to just caudal to the middle sagittal crest. Bone surface was exposed through dissection of the periosteum. Two standardized critical-size calvarial bone defects were created in each animal using a trephine (*d* = 5 mm). After removal of bone fragments, biomaterials were placed into the defect sites. At the final stage, the periosteum was retracted to cover the defect, and the skin incision was closed with interrupted PGA 3-0 sutures.

**Figure 1:**
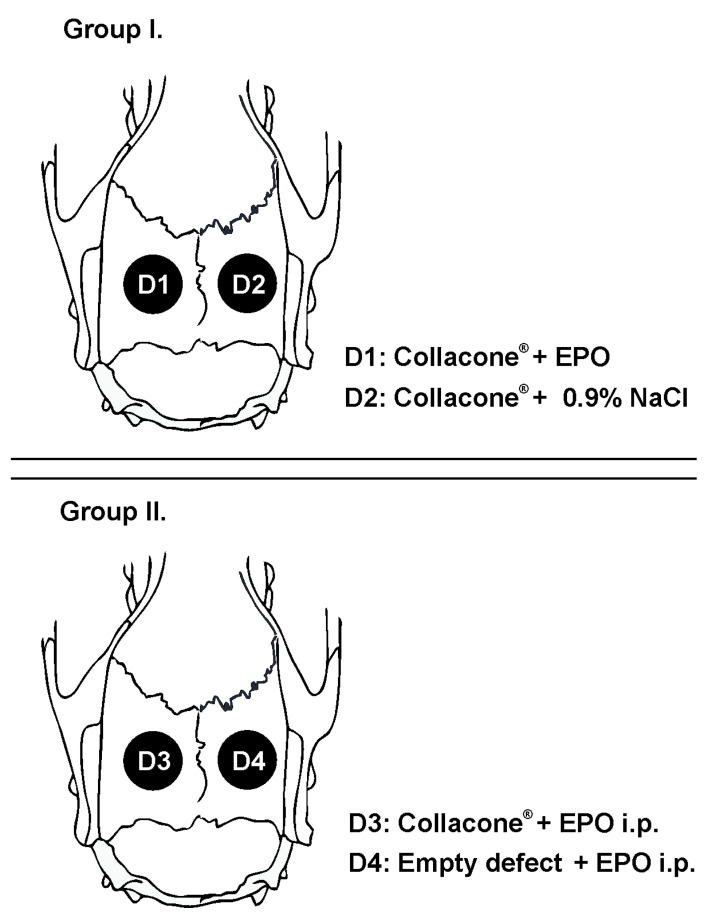
Experimental design—schematic presentation of groups and defects.

The rats were divided into two experimental groups. Depending on the type of biomaterial, four subgroups (D1, D2, D3, D4) were formed (Figure 1). In rats from experimental group I (*n* = 12), EPO was applied locally on a collagen cone in left defects, whereas a collagen cone soaked with physiological saline was placed in right defects. The rats from experimental group II (*n* = 12) were injected once intraperitoneally with 4900 IU/kg EPO; a collagen cone only was placed in left defects, whereas the right defects were left empty.

### 2.4. Clinical Evaluation and Blood Analysis

Daily physical examination was conducted in the postoperative period and special attention was paid to the operative wound state. Blood samples were collected on the day of surgery as well as on post-surgery days 30 and 90 from the lateral tail vein to determine the systemic effect of erythropoietin application. Erythrocyte counts (T/L), haemoglobin content (g/L) and haematocrit (%) were assayed on automated haemanalyzer (BS-5000 Vet, Mindray, Shenzhen, China).

### 2.5. X-ray Analysis

All rat heads were radiographed dorsoventrally with a stationary X-ray source, Philips Super 50 CP-D (Hamburg, Germany) at 46 KV and 8 mAs. Image J (ImageJ Analysis System, National Institutes of Health, Bethesda, Rockville, MD, USA) software was used to measure the radiopacity of each defect and surrounding bone, using a scale from 0 (black) to 250 (white). The relative bone density (RBD) was calculated by dividing the mean radiopacity of the defect by that of the surrounding bone.

### 2.6. CT Analysis

The bone regeneration course was also monitored by computed tomography (CT) scans at the same time intervals. For evaluation of the effect of erythropoietin administration, 3D images were reconstructed using the ANIMAGE reconstructing software (Fidex Animage 3825 Hopyard Road, LLC, Pleasanton, CA, USA). The images of maximum intensity generated by the software were used. By means of conventional 3D CT, evaluation of bone formation and bone bridging quality at the defect site was done by the 4-point scoring system of Patel et al., 2008 [29].

### 2.7. Statistical Analysis

Relative bone density values are presented as mean and standard deviation (SD), and Patel bone regeneration scores as median and range. Differences between relative bone density values of left and right defects were assessed by one-way analysis of variance ANOVA, whereas differences between bone regeneration scores and haematological parameters were assessed by the Mann-Whitney test. All analyses were performed with the statistical software MedCalc v.15.8 (MedCalc Software Ltd., Ostend, Belgium).

## 3. Results

### 3.1. Clinical and Haematological Examinations

There were neither complications in the postoperative period nor side effects from EPO application. The effect of EPO on blood levels is summarized in Table 1. In the group with local EPO application (group I), changes in erythrocyte counts, haemoglobin and haematocrit were insignificant, except for haemoglobin content which increased from 139 g/L on day 0 to 148 g/L at day 90 (*p* < 0.01).

All three parameters increased substantially and statistically significantly in the rats from the systemic EPO group. On day 30, significant changes were found in haemoglobin content (*p* < 0.05) and erythrocyte count (*p* < 0.001) vs. day 0, whereas on the 90th day, all three changed statistically significantly (*p* < 0.001).

The measured parameters on days 30 and 90 after local EPO application demonstrated the absence of any systemic effect of the preparation, as the values were within the reference ranges for the species.

### 3.2. X-ray Results

**Figure 2:**
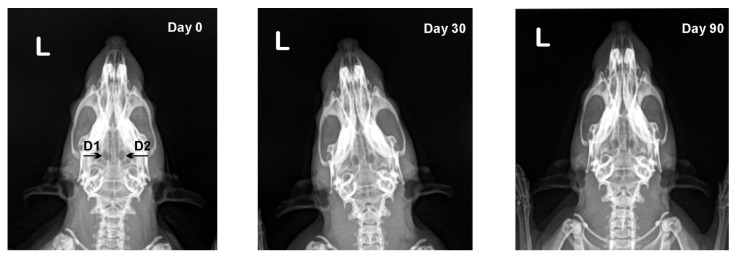
Serial dorsoventral radiographs of calvarial bones of rats from the first experimental group after local EPO on collagen cone (D1) and local physiological saline on collagen cone (D2) application on post-operative days 0, 30 and 90.

**Figure 3:**
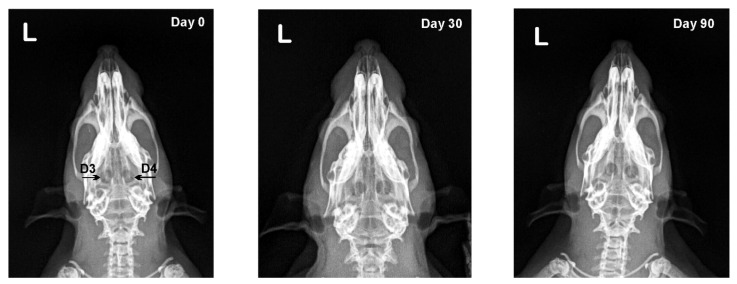
Serial dorsoventral radiographs of calvarial bones of rats from the second experimental group after collagen cone and i.p. EPO injection (D3) and empty defect and i.p. EPO injection (D4) on post-operative days 0, 30 and 90.

In both experimental groups, no radiologically-visible changes on operated calvarial bones were found until the end of the trial (Figure 2 and Figure 3).

In defects from the first and second subgroup (D1, D2) bone density increased with time, yet statistically insignificantly. The defects from the third and fourth subgroup (D3, D4) exhibited the opposite tendency, as relative bone density decreased (Table 2).

### 3.3. CT Results

**Figure 4:**
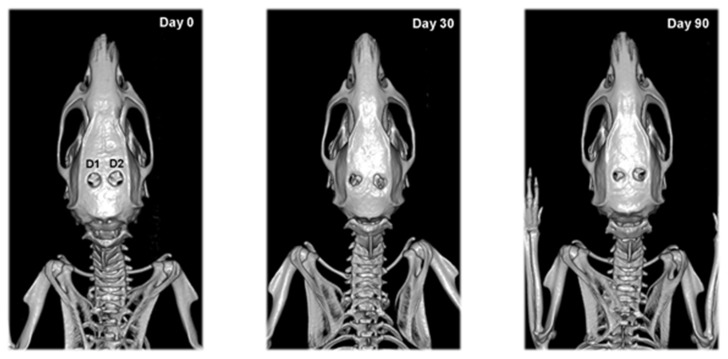
Serial 3D CT scans of calvarial bones of rats from the first experimental group after local EPO on collagen cone (D1) and local physiological saline on collagen cone (D2) application on post-operative days 0, 30 and 90.

On CT scans of calvarial bone defects from experimental group I, new bone formation appeared on defect margins and continued to grow until the end of the experiment (Figure 4). By the 30th day, there was a significant difference between Patel scores of defects treated locally with EPO vs. those treated with physiological saline (*p* < 0.05), whereas the scores on day 90 were the same (Table 3).

**Figure 5:**
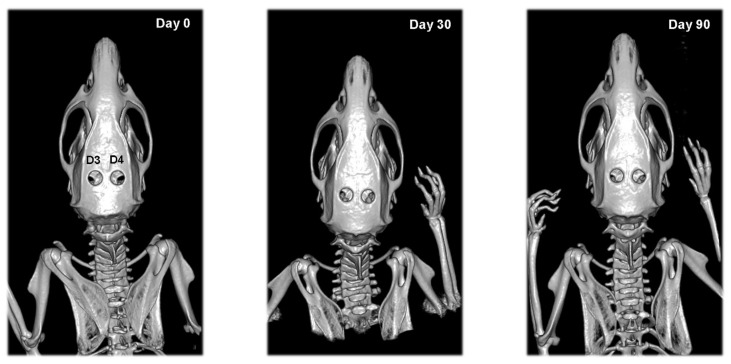
Serial 3D CT scans of calvarial bones of rats from the second experimental group after collagen cone and i.p. EPO injection (D3) and empty defect and i.p. EPO injection (D4) on post-operative days 0, 30 and 90.

Calvarial defects of rats from the second experimental group did not change their shape and area until the end of the study (Figure 5). Some individuals were an exception, showing bone formation at the site of the defect by the 90th day.

## 4. Discussion

The occurrence of large bone defects following different types of traumatic injuries, infection, advanced alveolar bone loss in periodontitis and peri-implantitis, tumor resection and skeletal abnormalities, or in cases of compromised bone regeneration including avascular necrosis and osteoporosis, require assisted skeletal regeneration in order to obtain good therapeutic results [30,31].

Osteoinduction and osteoconduction are the main mechanisms for stimulation of bone regeneration. Both processes were used and monitored in the present study. Osteoinduction engages undifferentiated and pluripotent cells which are stimulated to develop into the bone-forming cell lineage. One proposed definition is the process by which osteogenesis is induced [32]. This could be achieved through application of erythropoietin, which is supposed to act directly on bone marrow stromal cells (BMSCs) during their differentiation into osteoblasts. An indirect mechanism is also possible via activation through a signalling pathway for synthesis of bone morphogenic proteins (BMP). Their interaction with BMP-receptors results in differentiation of osteogenic progenitor cells into osteoblasts, and promotes callus formation [33]. The collagen cone used in the study has osteoconductive properties, acting as a scaffold for ingrowth and attachment of cells [34].

Hematological results demonstrated that systemic application of EPO increased statistically significantly erythrocyte counts, haemoglobin content and haematocrit. This fact was previously reported by Garcia et al. (2011) whose studies found a considerable difference in haemoglobin of mice injected with 500 IU/kg EPO over 5 weeks by the 2nd and 5th week vs. control levels [16]. Another experiment with rabbits injected on a daily basis with 250 IU/kg EPO over 20 days also reported increased haemoglobin and haematocrit values [22]. A higher peripheral red blood cell count, along with increased populations of haemopoietic and mesenchymal cells in the bone marrow, was confirmed by Sun et al. (2012) [35]. Obviously, both the low (yet higher than the physiological) EPO dose, as well as the single high dose, resulted in increased erythropoiesis.

Serial radiographs obtained in the course of our experiments for monitoring of bone regeneration evidenced that calvarial bone defects of both groups exhibited no radiologically-visible changes until the end of the study. This was probably due to the fact that in order to be visualized, the amount of callus should be substantial, and bone mineralization at least 40% [36].

Quantitative evaluation showed that relative bone density (RBD) of parietal bones increased in both groups, which was attributed to the newly-formed bone tissue along the periphery of the defect, confirmed histologically. Increased bone density after local application of 900 IU EPO on a collagen sponge was reported by Ahmed et al. (2019) in a rabbit experimental model. The authors also observed increased RBD in animals that received parenterally 4900 IU/kg EPO; however, this was not confirmed in our study. Furthermore, we observed the opposite tendency—radiologically decreased bone density, which could be attributed to occurring osteolysis on the margins of the defect [18]. According to White & Pharoah (2003), a digital radiography system could detect changes in bone tissue at a 5% demineralization level [36].

CT is considerably superior to classical radiography as it provides a much clearer and more detailed image of the head region. The ability of CT to create thinner sections and high-quality multiplanar reconstructions permits the earlier visualization of callus compared to radiography [37]. The CT results obtained in rats from experimental group I showed that local EPO application significantly enhanced bone formation during the first 30 days of osteogenesis. This was also observed by Bozlar et al. (2006), who found that EPO had a positive effect on the first stage of fracture healing. The authors noticed advanced angiogenesis, which explained the enhanced bone formation at the defect site, also confirmed by our histological finding [21].

Adequate blood supply is critical for bone regeneration, therefore EPO-induced neovascularization acts as an indirect pathway for stimulating bone healing by delivering oxygen and nutrients. Furthermore, the increased angiogenesis could enhance the callus mineralization rate [19]. Some authors believe that the mechanism by which EPO works is by influencing angiopoietin-1, while others report that treatment with rhEPO leads to the mobilization of CD34(+)/CD45(+) circulating progenitor cells in the peripheral blood and increases the number of functionally-active endothelial progenitor cells relevant to vasculogenesis [38].

According to our results, systemic EPO application did not result in bone formation dissimilar to previous reports. This could be due to the fact that we have chosen to apply a high single dose intraperitoneally, whereas other experimental designs have used a lower dose applied repeatedly for 5 to 7 days [21,39] or for 2–10 weeks [16,40]. There is still controversy regarding the appropriate dose and route of administration. In order to minimize animal stress from multiple injections, we aimed for the lowest possible dose and the shortest duration of treatment. Because of our unsatisfactory results, we assume that systemically-injected EPO does not reach target tissue in the optimal concentration to exert its positive effect on bone healing. Furthermore, recent research found that high EPO inhibits osteogenesis and adipogenesis, using transgenic mice overexpressing EPO (Tg6) [41].

Diagnostic imaging modalities in the present research are appropriate to prove the promising results regarding the local use of EPO for promoting bone regeneration in rat calvarial defects. However, more sophisticated investigation methods should be used to in order to explain the intimate mechanism of rhEpo effects on bone healing. Besides, systemic erythropoietin administration causes hematological changes that may result in side effects. Further studies analyzing long-term outcomes with regard to the safety of the drug will be needed before it can be clinically evaluated.

## 5. Conclusions

The evaluation of erythropoietin application for promoting bone regeneration in rat calvarial defects can be summarized as follows:

1. Single local dose rhEpo applied on collagen carrier could be used for enhancing bone healing without systemic effect.
2. Intraperitoneally-injected rhEpo did not result in bone formation. Simultaneously, it affects the erythropoiesis by increasing the erythrocyte counts, haemoglobin and haematocrit.
3. Computed tomography offers a much higher level of detail for visualizing changes during bone healing than conventional radiography.

## Figures and Tables

**Table 1:** Erythrocyte counts, haemoglobin content and haematocrit in rats from experimental groups I and II on day 0 (*n* = 24), 30 (*n* = 24) and 90 (*n* = 12). Values are presented as median (minimum–maximum range).

		Day 0	Day 30	Day 90
Erythrocyte count, T/L	Group I	6.22 (5.36–8.24)	6.74 (6.29–9.09)	6.77 (6.22–8.28)
	Group II	5.63 (5.03–8.00)	6.25 (5.66–7.04) ***	7.50 (7.22–7.87) ***
Haemoglobin content, g/L	Group I	139 (113–157)	142 (134–185)	148 (138–164) **
	Group II	117 (105–137)	142 (112–158) *	158 (150–163) ***
Haematocrit, %	Group I	34.2 (31.4–44.1)	37.6 (34.1–47.7)	37.9 (35.5–44.7)
	Group II	30.6 (26.9–36.0)	35.7 (30.5–42.0)	42.6 (38.8–44.1) ***

* *p* < 0.05; ** *p* < 0.01; *** *p* < 0.001 vs. Day 0 within each group.

**Table 2:** Relative bone density (mean ± standard deviation) of calvarial defects of rats on day 0 (*n* = 24), 30 (*n* = 24) and 90 (*n* = 12).

Defect	Day 0	Day 30	Day 90
D1	0.92 ± 0.02	0.92 ± 0.03	0.93 ± 0.03
D2	0.93 ± 0.02	0.93 ± 0.04	0.95 ± 0.04
D3	0.92 ± 0.02	0.91 ± 0.03	0.90 ± 0.03
D4	0.93 ± 0.01	0.92 ± 0.02	0.90 ± 0.02 *

D1: collagen cone + EPO; D2: collagen cone + 0.9% NaCl; D3: collagen cone; D4: empty defect; * *p* < 0.05 vs. Day 0 within each group.

**Table 3:** Bone regeneration scores according to Patel et al. (2008) on day of surgery (day 0, *n* = 24) and postoperative days 30 (*n* = 24) and 90 (*n* = 12). Values are presented as median and minimum–maximum range.

Defect	Day 0	Day 30	Day 90
D1	0 (0–0)	1 (0–2) **#	2 (1–2) **
D2	0 (0–0)	2 (2–3) ***	2 (2–3) ***
D3	0 (0–0)	0 (0–2)	0 (0–2)
D4	0 (0–0)	0 (0–2)	1 (0–2)

D1: collagen cone + EPO; D2: collagen cone + 0.9% NaCl; D3: collagen cone; D4: empty defect; ** *p* < 0.01; *** *p* < 0.001 vs. Day 0 within a group; # *p* < 0.05 between D1 and D2 scores.

## Data Availability

Data can be obtained from the corresponding author upon reasonable request.
